# Commentary: Tranexamic Acid in Patients Undergoing Coronary-Artery Surgery

**DOI:** 10.3389/fcvm.2017.00045

**Published:** 2017-07-17

**Authors:** Xiang-Dong Wu, Ke-Jia Hu, Wei Huang

**Affiliations:** ^1^Department of Orthopaedic Surgery, The First Affiliated Hospital of Chongqing Medical University, Chongqing, China; ^2^Department of Neurosurgery, Massachusetts General Hospital, Harvard Medical School, Boston, MA, United States; ^3^Department of Microsurgery, Huashan Hospital, Fudan University, Shanghai, China

**Keywords:** tranexamic acid, seizures, thromboembolic complications, coronary-artery surgery, dose

Tranexamic acid has been widely and routinely used as a prophylactic treatment to reduce the rate and severity of blood loss in many surgical disciplines, such as orthopedic and cardiac surgical procedures. However, whether such therapy would increase the risk of arterial or venous thrombotic events has long been uncertainty (1). Myles et al. performed the ATACAS trial published in *NEJM*, which underscored the benefits and potential risks of tranexamic acid in coronary-artery surgery (2). Tranexamic acid is associated with a lower risk of bleeding, without a higher risk of death or thrombotic complications, meanwhile, also higher postoperative seizures risk. This commentary aimed to discuss several issues related to tranexamic acid and the trial itself.

First, their work highlighted that the optimal dose and dosing regimens of tranexamic acid are still issues. On the one hand, their excellent work confirmed the efficacy of tranexamic acid to decrease blood loss, which is undisputed (3) and a definitive conclusion has been drawn from the literature (1). However, the increased incidence of seizures is not trivial. Indeed, seizures have been regarded as an adverse event of tranexamic acid for some time. Previous studies have confirmed a dose-dependent relationship between the dose of tranexamic acid and incidence of seizures (4). A recent work compared the molecular structure of glycine with antifibrinolytic drugs and revealed a novel mechanism that tranexamic acid inhibits glycine receptors which explained why seizures increased after cardiovascular surgery (5). Accordingly, intravenous administration of high-dose tranexamic acid of 50 mg/kg and above should be deliberative, particularly in elder patients or patients who suffering from renal impairments (6). However, previous studies have indicated that high dose of tranexamic acid was more effective than low dose (7). It seems that we are trapped in a dilemma. Fortunately, multiple boluses of tranexamic acid have been proved to be superior to single boluse (8), hence, multiple boluses of low dose of tranexamic acid should be preferred and recommended as an alternative regimen to avoid tranexamic acid-induced seizures, and the optimal regimens warrant further investigation.

Second, Myles et al. considered that intravenous administration of tranexamic acid is not exempt from vascular accidents, the relationship of postoperative seizures with stroke and death observed in this trial suggests a possible underlying thromboembolic cause of the seizures. An observational study found that tranexamic acid was associated with an increased risk of venous thromboembolism, although the risk estimate did not reach statistical significance (9). Therefore, a growing tendency of thrombogenesis was strongly suspected even if no statistical significant differences were found regarding the risk of death or thrombotic complications. Noticeably, thrombotic event is extremely rare, thus question remains that no significant difference in thrombotic complications does not indicate no difference, or no changes in coagulability after intravenous of tranexamic acid.

Third, cardiac surgery would induce multifactorial changes in coagulability (10), while standard coagulation measurements have limited values as they just reflect deficiencies in procoagulant factors, without balancing concurrent deficiencies of anticoagulant factors such as thrombomodulin (11). Indeed, there has been a persistence of hypercoagulability state after cardiac surgery, and thromboelastography would be a preferable diagnostic assay to evaluate the accurate hemostasis and fibrinolysis (12). Additional perioperative coagulation assessment with thromboelastography might contribute to predict and detect the trend of the dynamic variation of coagulability, distinguish enzymatic hypercoagulability from platelet hypercoagulability or mixed hypercoagulability (13), which might not only reflect changes in coagulability and underlying prothrombotic tendency but also conducive to interpreting no significant differences in thrombotic events but in seizures. Regrettably, perioperative coagulation assessment was absent despite blood samples were collected (2). Marginal increase in hypercoagulability may not induce severe thrombotic complications, but perhaps relates to reduced cerebral blood flow and increased risk of cerebral infarction. One study reported ischemic strokes occurred after administration of tranexamic acid with a particular genotype, which indicated tranexamic acid may induce clinical seizures in susceptible patients (14) as a confounding factor.

One more concern is the primary outcome of the trial was a composite of death and a couple of thrombotic events. The investigators ignored the validity of composite end points. The present study incorporated component end points with heterogeneous importance to patients, the incidence of important components is small, and treatment effects differ significantly across components, which would be misleading when interpreting the results (15). If pulmonary embolism was the primary outcome, the required sample size would be more enormous; therefore, adverse events with low incidences were not ideal indicators.

In summary, current studies alerts and warns us that administration of high-dose of tranexamic acid needs discretion, multiple boluses of low-dose of tranexamic acid should be preferred and recommended as an alternative regimen in CABG, and future trials are warrant since no unanimous agreement reached and concerns remain regarding the optimal dosage and frequency of tranexamic acid. Tranexamic acid might induce hypercoagulability although no significant difference was detected in thrombotic complications, and thromboelastography would be a better detection method. The composite of the primary outcome in the results section of the trial is debatable. Whether these findings are relevant to other patient populations such as arthroplasty, requires further investigation.

## Author Notes

*Guarantor of article*: WH is acting as the submission’s guarantor. Address: The First Affiliated Hospital of Chongqing Medical University, No. 1, Youyi Road, Yuanjiagang, Yuzhong District, Chongqing, 400016, China; Telephone number: (+86) 13883383330, Fax number: (+86) 023 89011212, E-mail: drhuangwei68@gmail.com. *Submission statement*: We confirm that this manuscript has not been published elsewhere and is not under consideration by another journal. All authors have approved the manuscript and agreed with submission to *Frontiers in Cardiovascular Medicine*.

## Author Contributions

X-DW contributed substantially to conception and design and acquisition, analysis, and interpretation of data; drafted the article; gave final approval of the version to be published; and agreed to act as guarantor of the work. K-JH contributed substantially to acquisition, analysis, and interpretation of data; drafted the article; gave final approval of the version to be published; and agreed to act as guarantor of the work. WH contributed substantially to conception and design and acquisition, analysis, and interpretation of data; revised it critically for important intellectual content; gave final approval of the version to be published; and agreed to act as guarantor of the work.

## Conflict of Interest Statement

The authors declare that the research was conducted in the absence of any commercial or financial relationships that could be construed as a potential conflict of interest.
